# Characterization of adult T-cell leukemia/lymphoma patients with specific skin lesions in a tertiary dermatological service in Brazil

**DOI:** 10.3389/fmed.2025.1505865

**Published:** 2025-02-06

**Authors:** Mariana Valente, José Antonio Sanches, Youko Nukui, Jade Cury-Martins, Bruno Castro Souza, Juliana Pereira, Denis Miyashiro

**Affiliations:** ^1^Department of Dermatology, University of São Paulo, São Paulo, Brazil; ^2^Discipline of Hematology, University of São Paulo, São Paulo, Brazil; ^3^University of São Paulo, São Paulo, Brazil; ^4^Department of Dermatology, University of São Paulo Medical School, São Paulo, Brazil

**Keywords:** adult T-cell leukemia-lymphoma, cutaneous T-cell lymphoma, HTLV-1 infections, HTLV-1, cohort

## Abstract

**Introduction:**

Human T-lymphotropic virus type-1 (HTLV-1) is endemic in some countries, including Brazil. HTLV-1 is the etiological agent of adult T-cell leukemia-lymphoma (ATLL), a rare and aggressive CD4+ T-lymphocyte malignancy. ATLL affects 1–5% of virus carriers. Dermatological involvement occurs in 40–70%. Diagnosis is based on clinicopathologic correlation and HTLV-1 serology. There are few therapeutic options so far.

**Methods:**

This is an observational retrospective cohort study with ATLL patients followed in a tertiary hospital in São Paulo, Brazil. Data were collected at diagnosis. Survival curves using the Kaplan–Meier method were analyzed with log-rank test, univariate and multivariate analyses were performed with the Cox proportional hazards model.

**Results:**

Forty-four patients were studied, 24 females (54.5%), and 20 males (45.5%). The median age at diagnosis was 59.4 years. Classification at diagnosis was: 16 (36.4%) chronic (93.7% unfavorable, 6.2% favorable), 14 (31.8%) acute, 10 (22.7%) smoldering, four (9.1%) lymphoma, and none with primary cutaneous tumoral. Regarding skin lesions, 18 (40.9%) had plaques; 15 (34.1%) nodules/tumors; 11 (25.0%) papules; 10 (22.7%) erythroderma; seven (15.9%) patches; two (4.5%) ichthyosis; one (2.3%) purpuric lesions. Epidermotropism/exocytosis of lymphocytes was observed in 25 patients (62.5%), and Pautrier microabscesses in three (7.3%). Four patients (10.0%) had subcutaneous involvement, two (5.0%) folliculotropism, two (5.0%) angiocentrism, and one (2.5%) perineural involvement. Ten patients (25.0%) presented a lichenoid pattern. Thirty-four patients (79.1%) had increased lactate dehydrogenase; 20 (45.5%) lymphocytosis; six (13.6%) flower cells in peripheral blood; six (14.6%) hypercalcemia; five (12.2%) hypoalbuminemia. Beta-2 microglobulin was increased in all 24 cases investigated. Monoclonal T-lymphocytes were observed in the blood of 23 patients (76.7%) and the skin of 19 (76.0%). Thirty patients (68.2%) died. Median overall survival was 32.3 months. Acute and chronic unfavorable forms had worse prognoses, with median overall survival of 23.3 and 34.1 months, respectively (*p* = 0.0011). After multivariate analysis, Shimoyama classification (acute) and urea levels were associated with poorer prognoses.

**Conclusion:**

We described a large Brazilian cohort of ATLL with cutaneous involvement. Description of clinical, pathology, laboratory, and follow-up data, and factors associated with poorer survival is essential to provide better care and to improve the quality of life of these patients.

## 1 Introduction

### 1.1 HTLV-1 virus and transmission

Human T-lymphotropic virus type 1 (HTLV-1) is an enveloped single-stranded RNA (ribonucleic acid) virus of the *Retroviridae* family and *Oncovirinae* subfamily that affects approximately 10 to 20 million people worldwide and is responsible for the development of adult T-cell leukemia/lymphoma (ATLL). Infection is mainly the result of transmission of infected lymphocytes or, less commonly, of free viral particles through breastfeeding, transfusion of contaminated blood or blood products, sharing of contaminated syringes, and unprotected sexual intercourse (1–3). HTLV-1 exhibits tropism for CD4 T cells, but can also infect dendritic cells. The glucose transporter, glut-1, has been identified as a receptor for the HTLV-1 envelope glycoprotein, encoded by a gene known as env (4). HTLV-1-infected CD4 T cells can produce CCL22 (C-C chemokine ligand 22), a ligand for CCR4 (C-C chemokine receptor 4). CCL22 attracts CCR4-expressing CD4 cells, resulting in preferential transmission of HTLV-1 to CCR4-positive and CD4-positive T-cells (5). After entering the cell, HTLV-1’s RNA undergoes reverse transcription, generating a DNA (deoxyribonucleic acid) product that integrates into the host genome. After integration, viral replication can occur in two ways: through reexpression of the integrated provirus, which forms a new enveloped virion that infects other cells (also called “infectious” replication). Or, in another way, when the integrated provirus replicates at each mitotic cell division. This results in a low rate of viral replication and high transcription fidelity, which explains the high genetic stability of HTLV-1 (6).

The virus regulates its transcription through two pathways: via Tax, which acts as a transcriptional activator, and via Rex, which acts to suppress viral transcription (7, 8). The opposing actions of Tax and Rex result in the transient expression of viral gene products, which may aid in evading the host immune response (9). When integrated as a provirus, persistent infection is established and subsequent translation of viral products can induce numerous cellular alterations favoring the survival and cellular proliferation of the virus (7–9).

### 1.2 Conceptual and epidemiological aspects of ATLL

In 1977, Japanese researchers first described ATLL, characterized by a rare and potentially aggressive mature T-cell neoplasm that affects the lymphatic, hematologic, and/or skin systems and was later associated with HTLV-1 infection (1, 10).

ATLL is a non-Hodgkin lymphoma that can undergo leukemic progression and presents several clinical characteristics. It is classified as cutaneous T-cell lymphoma by the World Health Organization (WHO) and the European Organization for Research and Treatment of Cancer (EORTC), which proposed a classification for primary cutaneous lymphoproliferative processes (11).

The prevalence of ATLL varies according to the prevalence of HTLV-1. Infection by the virus is endemic in some regions of the world, such as Southwest Japan, Iran, Central and Western Africa, and regions of Central and South America (3). Nonetheless, the incidence in non-endemic areas has been increasing due to migration phenomena in recent decades (12). In Brazil, it is estimated that approximately 800,000 people are infected with HTLV-1 and the state of Bahia (located in the Northeast) has the highest prevalence of individuals affected by the virus (0.48%), where the population is largely composed of African descendants (13–15). However, the prevalence of the infection is also high throughout other states of the Northeast and North regions (14). In Brazil, as the infection is not subject to mandatory reporting, the available epidemiological data come from regional population studies conducted on blood donors, pregnant women, injecting drug users, and sex workers (15).

Infection by the virus may be asymptomatic in most cases, but it is estimated that 5 to 10% of those infected will present clinical manifestations, such as HTLV-1-associated myelopathy/tropical spastic paraparesis (HAM/TSP), ATLL, infective dermatitis, and other pulmonary, ophthalmological, urological, intestinal and joint manifestations (3).

ATLL, in turn, is observed almost exclusively in adults and is very rare in children due to the long latency of the viral infection necessary for the development of the disease. The average age at diagnosis is between 50 and 60 years, with a slight male predominance (3, 16). It is known that after infection by the virus, approximately 20 years of latency are required for the development of ATLL, which occurs in only approximately 1–5% of infected individuals (3, 17). In addition, exposure to the virus early in life increases the risk of developing the disease, as does the use of immunosuppressants for other conditions (18).

### 1.3 Clinical aspects, classification, and prognosis

ATLL may present with manifestations in the peripheral blood, lymph nodes, skin, and, less commonly, involvement of the bone marrow, central nervous system (CNS), and respiratory tract (19). Clinically, patients are classified into four variants according to the Shimoyama criteria (Table 1): acute (leukemic), lymphoma, chronic (later also subdivided into favorable and unfavorable), and smoldering (20).

**Table 1:** Shimoyama classification (20, 28).

	Acute	Lymphoma	Chronic	Smoldering
General characteristics				
Frequency	55%	20%	20%	5%
Average survival	6 months	10 months	24 months	-
4-year survival	5%	6%	27%	66%
Diagnostic criteria				
Anti-HTLV-1	Yes	Yes	Yes	Yes
Lymphocytes	ND	<4.000/mL	≥4.000/mL	<4.000/mL
Lactate dehydrogenase	ND	ND	<2 times upper limit of normal	<1,5 times upper limit of normal
Corrected calcium	ND	ND	<5,5 mEq/L	<5,5 mEq/L
Abnormal T lymphocytes	5%*	<1%	5%*	5%*
Flower cells	Yes	No	Occasionally	Occasionally
Tumors				
Skin and/or lung	ND	ND	ND	If abnormal lymphocytes <5%*
Lymph nodes	ND	Yes	ND	No
Liver or spleen	ND	ND	ND	No
Central nervous system	ND	ND	No	No
Bone	ND	ND	No	No
Ascite	ND	ND	No	No
Pleural effusion	ND	ND	No	No
Gastrointestinal tract	ND	ND	No	No

ND = non-defining. *If < 5% of abnormal T lymphocytes, histological proof of the tumor lesion is required.

More recently, an additional, slowly progressive form presenting exclusively with cutaneous lesions, has been described, called primary cutaneous tumoral (PCT) (21).

Clinical presentations of ATLL are related to the prognosis of the disease. Acute (leukemic), lymphoma and chronic unfavorable forms are considered aggressive, while the chronic favorable and smoldering forms are considered indolent (19). It is also important to emphasize that all presentations of ATLL may evolve to the acute form, characterizing the leukemization of the disease (20). The acute variant has a poor prognosis and a mean survival of approximately 6 months. In these cases, patients most frequently present systemic symptoms, hepatosplenomegaly and adenomegaly, pronounced elevation of lactate dehydrogenase (LDH) and leukocytes, as well as the presence of circulating malignant cells known as flower cells due to greater involvement of the bone marrow. In addition, the presence of hypercalcemia with or without lytic bone lesions is also very common. Pulmonary and CNS involvement, evidenced by the presence of infiltrates and masses on imaging tests may also occur. The lymphoma subtype is characterized by significant adenomegaly without pronounced blood involvement. Patients often have high LDH levels and may have hypercalcemia. The prognosis is also poor, with a mean survival of 10 months (19, 20, 22–26). The chronic variant can be subdivided into favorable and unfavorable according to laboratory data (increased urea, hypoalbuminemia and increased LDH are diagnostic criteria for the unfavorable chronic form). Patients most frequently present with skin lesions, lymphadenopathy, leukocytosis, and absolute lymphocytosis that may remain stable for months to years. In the chronic favorable form, the mean survival is two to five years, while in the unfavorable form, the prognosis is poor and similar to that of the acute and lymphoma-type variants. In the smoldering variant, patients are often asymptomatic, except for occasional skin and/or lung lesions. The median survival in these cases is approximately three to five years (26). Finally, the PCT variant is a distinct subtype with skin lesions that appear as rapidly growing tumors with pathologic findings characterized by a high-grade pleomorphic T-cell lymphoma with medium or large cells, prominent perivascular infiltration, and scant epidermotropism. This variant has an intermediate course, not as aggressive as the acute and lymphoma forms, but not as indolent as the chronic and smoldering forms (21).

It is also important to emphasize that patients with ATLL have an immunosuppressed immune state, at risk of developing opportunistic infections such as *Pneumocystis jirovecii* pneumonia, cryptococcal meningitis, and disseminated herpes zoster, in addition to disseminated strongyloidiasis, which may be severe and fatal (27).

Cutaneous manifestations of ATLL are present in 40–70% of patients and are more frequent in chronic and smoldering forms, although they may affect any clinical subtype. In addition, skin lesions may be the first manifestation of the disease (hence the importance of recognizing them) or appear after other signs and symptoms. Lesions may be further subdivided into specific (represented by neoplastic infiltrate in histology) or nonspecific (composed of inflammatory cell infiltrate) (28).

Specific lesions present as patches and plaques similar to mycosis fungoides (MF) lesions, papules, nodules and tumors, erythroderma (erythema and scaling covering ≥80% of the body surface area), and purpuric lesions. Xerosis and acquired ichthyosis are frequently present in patients and can be considered as a nonspecific or specific manifestation of ATLL (28, 29). The type of skin lesion has also been associated with the prognosis, with shorter survival in patients with nodular-tumoral lesions or erythroderma (30).

### 1.4 Pathological and immunological aspects

The histopathology of ATLL skin lesions is highly variable and has characteristics that overlap with other cutaneous T-cell lymphomas not associated with HTLV-1. The histological findings may be similar to those of MF, anaplastic large cell lymphoma (ALCL), or peripheral T-cell lymphoma unspecified (PTCL-U). In MF, the main histological aspects are the presence of epidermotropism, Pautrier microabscesses (atypical lymphocytes grouped around a Langerhans cell), and cells with cerebriform nuclei. In ALCL, on the other hand, the neoplastic cells are generally large and cohesive, with abundant cytoplasm and large, rounded nuclei with a single central nucleolus, diffusely affecting the dermis. In PTCL-U, a wide variation in cell size and marked nuclear pleomorphism are characteristically observed. In indolent forms, the infiltrate may be sparse and with little atypia (19).

Immunohistochemistry (IHC) is characterized by positivity for CD3, CD4, and CD5; negativity for CD8 and CD7. CD25 is frequently positive, but it is not specific. In some cases, especially in those with ALCL morphology, CD30 may also be positive. In addition, the Ki-67 proliferative index represents a relevant factor for the prognostic evaluation (31).

It is also important to emphasize that the most characteristic cell of ATLL is observed in the peripheral blood smear, especially in leukemic cases (acute form). These are medium-sized lymphocytes with condensed chromatin and bizarre multilobed nuclei, known as flower cells (32).

### 1.5 Diagnosis

The diagnosis of ATLL is made according to the presence of clinical manifestations, histology, immunophenotypic, and molecular characteristics (monoclonality) of malignant cells, in addition to confirmation of HTLV-1 infection (33). Detection of HTLV-1 infection is most commonly performed by identifying antibodies against the virus through enzyme-linked immunosorbent assay (ELISA) for screening and Western blotting (WB) for confirmation and distinction of infection between HTLV-1 and 2. Tests based on polymerase chain reaction (PCR) to detect proviral DNA in tumor cells should be performed in the rare cases in which serology is negative, but suspicion of ATLL is high, such as in patients with immunodeficiencies in which antibody production is compromised. In atypical or doubtful cases, it is essential to search for the proviral integration test in tumor cells, however, this test has very low availability in Latin America, being performed only in a few research laboratories (19, 33, 34).

### 1.6 Treatment

Treatment of ATLL is still limited and varies according to the classification and medications available in different countries. In indolent forms with skin lesions, therapeutic options include “watch and wait” and skin-directed therapies (topical corticosteroids and phototherapy), which have anti-inflammatory and antiproliferative effects on the skin. Radiotherapy, arsenic trioxide (ATO), and a combination of interferon-alpha (IFN) with zidovudine (AZT) may also be necessary (33, 35).

In aggressive forms, IFN and AZT are also used, but chemotherapy may be necessary. Hematopoietic stem cell transplantation (HSCT) is a potentially curative option and may be a possibility for more severe cases, but with a high incidence of complications and mortality related to the transplantation itself (33, 35).

Regarding chemotherapy, the following regimens are primarily used: CHOP (cyclophosphamide, doxorubicin, vincristine, and prednisone), CHOEP (cyclophosphamide, doxorubicin, etoposide, vincristine, and prednisone), and Hyper-CVAD (course A: cyclophosphamide, doxorubicin, vincristine, dexamethasone; course B: methotrexate, cytarabine) (33, 35). In Japan, the VCAP-AMP-VECP regimen (vincristine, cyclophosphamide, doxorubicin, prednisone; doxorubicin, ranimustine, prednisone; vindesine, etoposide, carboplatin, prednisone) appears to be slightly more effective than traditional regimens (36). Other drugs have been used to treat ATLL, including mogamulizumab (a humanized anti-CCR4 monoclonal antibody) (37, 38), brentuximab vedotin (a chimeric anti-CD30 monoclonal antibody associated with the antimicrotubule agent monomethyl auristatin E) (39) and lenalidomide (a thalidomide analog) (40).

Treatment should be continued for life. However, many patients are resistant to treatment or progress even after a long period of disease control. There is a lack of prospective and large-scale studies in the literature to establish more effective therapeutic strategies (33, 35).

In addition to geographic variations in the treatments used, studies show that the genetic profile varies between Japanese and Western cases (41). Thus, a better understanding of the clinical, histological, immunological, and follow-up characteristics of ATLL in different countries is essential for developing and applying new therapeutic strategies.

We aimed to describe and analyze the characteristics of ATLL patients with specific skin lesions followed in a tertiary dermatological service in Brazil.

## 2 Methods

This retrospective and non-concurrent cohort study was approved by the Ethics Committee of the University of São Paulo.

All patients diagnosed with ATLL with specific skin lesions followed at the Cutaneous Lymphoma Outpatient Clinic of the Division of Clinical Dermatology at the University of São Paulo Medical School between January 1989 and December 2023 were included.

The clinical, laboratory, immunopathological, molecular, and imaging data at the time of diagnosis were collected from medical records. The final diagnosis was established based on the correlation of clinical, serological, histopathological, immunophenotypic, and molecular data. Cases in which the diagnosis was not well established were excluded from the analysis.

### 2.1 Clinical and demographic data

Demographic data were collected (gender, age at diagnosis, place of birth, and self-declared ethnicity). Clinical features, such time of the current disease, and the date of diagnosis were considered. We collected information regarding comorbidities and other neoplasms.

Skin lesions were described according to their morphology: patches, plaques, papules, nodules/tumors, erythroderma, purpuric lesions, ichthyosiform lesions, or other less commonly described skin lesions.

Lymph nodes of the cervical, axillary, and inguinal chains were considered clinically suspicious if: size ≥1.5 cm in the largest diameter, hardened and irregular consistency, fixed, or forming a conglomerate of lymph nodes.

### 2.2 Histopathology and immunophenotypic data of the skin

Data from the analysis of the cutaneous anatomopathological material were described in terms of the presence of atypical lymphocytic infiltrate, presence or absence of lymphocyte exocytosis and/or epidermotropism, Pautrier microabscesses, pattern of dermal/subcutaneous involvement, immunophenotype of the malignant cells (CD3, CD4, CD8, CD7, CD25, CD30, Ki-67), final diagnosis after histopathological and immunohistochemical analysis.

### 2.3 Laboratory data

Regarding systemic involvement, the extracutaneous dissemination of the lymphoproliferative process was assessed. Lymph node involvement was considered when a suspicious lymph node observed clinically or by imaging methods (CT or PET) was detected, with confirmation of histological involvement by lymph node biopsy. Laboratory tests included complete blood count, serum levels of LDH, urea, albumin, calcium, beta-2-microglobulin (B2M), serology for HIV and HTLV-1 (if ELISA was positive, confirmation was performed with Western Blot), search for atypical cells in peripheral blood smears, immunophenotyping of lymphocytes by flow cytometry in peripheral blood, and search for T-cell receptor (TCR) gene rearrangement by polymerase chain reaction (PCR) in peripheral blood and skin.

### 2.4 Classification, evolution, and prognosis

Stratification regarding the form of presentation of ATLL at the time of diagnosis was performed according to the Shimoyama classification, including the PCT form.

Treatments performed throughout clinical follow-up were considered.

Data regarding the date of the last evaluation or death were checked. Regarding the patient’s status, the following were considered, according to oncology protocols: patient alive disease-free (AWoD), alive with evidence of disease (AWD), dead for another case (DOC), and dead of disease (DOD). Survival was analyzed considering the date of diagnosis, follow-up time, and patient status at the last evaluation.

### 2.5 Statistical analysis

Shapiro–Wilk test of normality demonstrated that all quantitative variables were nonparametric. Thus, they were described using median and 25 and 75% quartiles. Regarding qualitative variables, proportions, and percentages were described. The association between quantitative variables and the different groups analyzed was performed using the Wilcoxon Rank-sum test or the Kruskal-Wallis test with Dunn’s multiple comparisons post-test. The association between the qualitative variables and the different groups analyzed was performed using the chi-square test or Fisher’s exact test. The survival curves were plotted according to the Kaplan–Meier method. The difference between the median overall survival of the groups analyzed was assessed using the Cox proportional hazards test and the log-rank test. All statistical analyses were performed using STATA 13.0 software (STATA Corp. Texas, United States). Statistical significance was considered when the *p*-value was less than or equal to 0.05.

## 3 Results

### 3.1 Case series

Between January 1989 and December 2023, 44 patients with ATLL and specific skin lesions were evaluated at our service. There were 16 patients (36.4%) with the chronic form (15 or 93.7% unfavorable and one or 6.2% favorable chronic ATLL); 14 patients (31.8%) with acute; 10 patients (22.7%) with smoldering; and four (9.1%) with lymphoma subtype. There was no patient with the primary cutaneous tumoral subtype of ATLL (Table 2).

**Table 2:** Demographic, clinical, laboratory, and follow-up data according to ATLL subtype.

	Acute	CU*	CF**	Lymphoma	Smoldering	Total	*P*-value
N (%)	14 (31.8)	15 (34.1)	1 (2.3)	4 (9.1)	10 (22.7)	44	-
Median age in years (IQR^#^)	53.9 (43.3–63.4)	61.2 (56.9–71.2)	30.9	71.9 (63.1–79.6)	60.4 (34.2–64.8)	59.4 (47.7–66.4)	0.0456
HTLV-1-associated comorbidities	1 HAM^##^	2 ID^§^	0	0	3 HAM^##^/1 ID^§^	4 HAM^##^/3 ID^§^	-
Patches (%)	0/14 (0.0)	2/15 (13.3)	0/1 (0.0)	1/4 (25.0)	4/10 (40.0)	7/44 (15.9)	0.075
Plaques (%)	6/14 (42.9)	4/15 (26.7)	1/1 (100)	1/4 (25.5)	6/10 (60.0)	18/44 (40.9)	0.326
Papules (%)	5/14 (35.7)	5/15 (33.3)	0/1 (0.0)	0/4 (0.0)	1/10 (10.0)	11/44 (25.0)	0.466
Nodules/tumors (%)	6/14 (42.9)	4/15 (26.7)	0/1 (0.0)	3/4 (75.0)	2/10 (20.0)	15/44 (34.1)	0.280
Erythroderma (%)	3/14 (21.4)	4/15 (26.7)	0/1 (0.0)	1/4 (25.0)	2/10 (20.0)	10/44 (22.7)	0.974
Purpuric lesions (%)	1/14 (7.1)	0/15 (0.0)	0/1 (0.0)	0/4 (0.0)	0/10 (0.0)	1/44 (2.3)	0.659
Other types of lesions (%)	0/14 (0.0)	5/15 (33.3)	0/1 (0.0)	0/4 (0.0)	3/10 (30.0)	8/44 (18.2)	0.416
Lymph node enlargement detected clinically or by imaging studies	10/14 (71.4)	8/15 (53.3)	0/1 (0.0)	4/4 (100)	6/10 (60.0)	28/44 (63.6)	0.298
Leukocytosis (%)	8/14 (57.1)	12/15 (80.0)	1/1 (100)	0/4 (0.0)	2/10 (20.0)	23/44 (52.3)	0.005
Lymphocytosis (%)	9/14 (64.3)	13/15 (86.7)	1/1 (100.0)	0/4 (0.0)	0/10 (0.0)	23/44 (52.3)	<0.001
Increased LDH (%)	14/14 (100)	15/15 (100)	0/1 (0.0)	2/4 (50.0)	3/9 (33,3)	34/43 (79.1)	<0.001
Hypercalcemia (%)	6/13 (46.1)	0/14 (0.0)	0/1 (0.0)	0/4 (0.0)	0/9 (0.0)	6/41 (14.6)	0.004
Hypoalbuminemia (%)	1/14 (7.1)	2/14 (14.3)	0/1 (0.0)	1/4 (25.0)	1/8 (12.5)	5/41 (12.2)	0.912
Increased Beta-2 microglobulin (%)	9/9 (100)	9/9 (100)	1/1 (100)	(100)	4/4 (100)	24/24 (100)	-
Flower cells (%)	5/14 (35.7)	0/15 (0.0)	0/1 (0.0)	1/4 (25.0)	0/10 (0.0)	6/44 (13.6)	0.035
Monoclonal population of T-cells in the blood (%)	9/9 (100)	11/12 (91.7)	1/1 (100.0)	2/3 (33.3)	0/5 (0.0)	23/30 (76.7)	<0.001
Monoclonal population of T-cells on the skin (%)	7/7 (100)	7/9 (77.8)	ND^+^	3/3 (100)	2/6 (33.3)	19/25 (76.0)	0.045
Deaths (%)	11/14 (78.6)	10/15 (66.7)	1/1 (100)	3/4 (75.0)	5/10 (50.0)	30/44 (68.2)	0.508
Median overall survival (months)	23.3	34.1	29.6	53.5	128.4	32.3	0.0011

Number of cases = 44. *CU: Chronic unfavorable; **CF: Chronic favorable; ^+^ND: Not done; ^#^IQR: Interquartile range; ^##^HAM: HTLV-1-associated myelopathy; ^§^ID: Infective dermatitis.

### 3.2 Demographic data

Demographic, clinical, laboratory, and follow-up data are summarized in Table 2.

**GRAPH 1:**
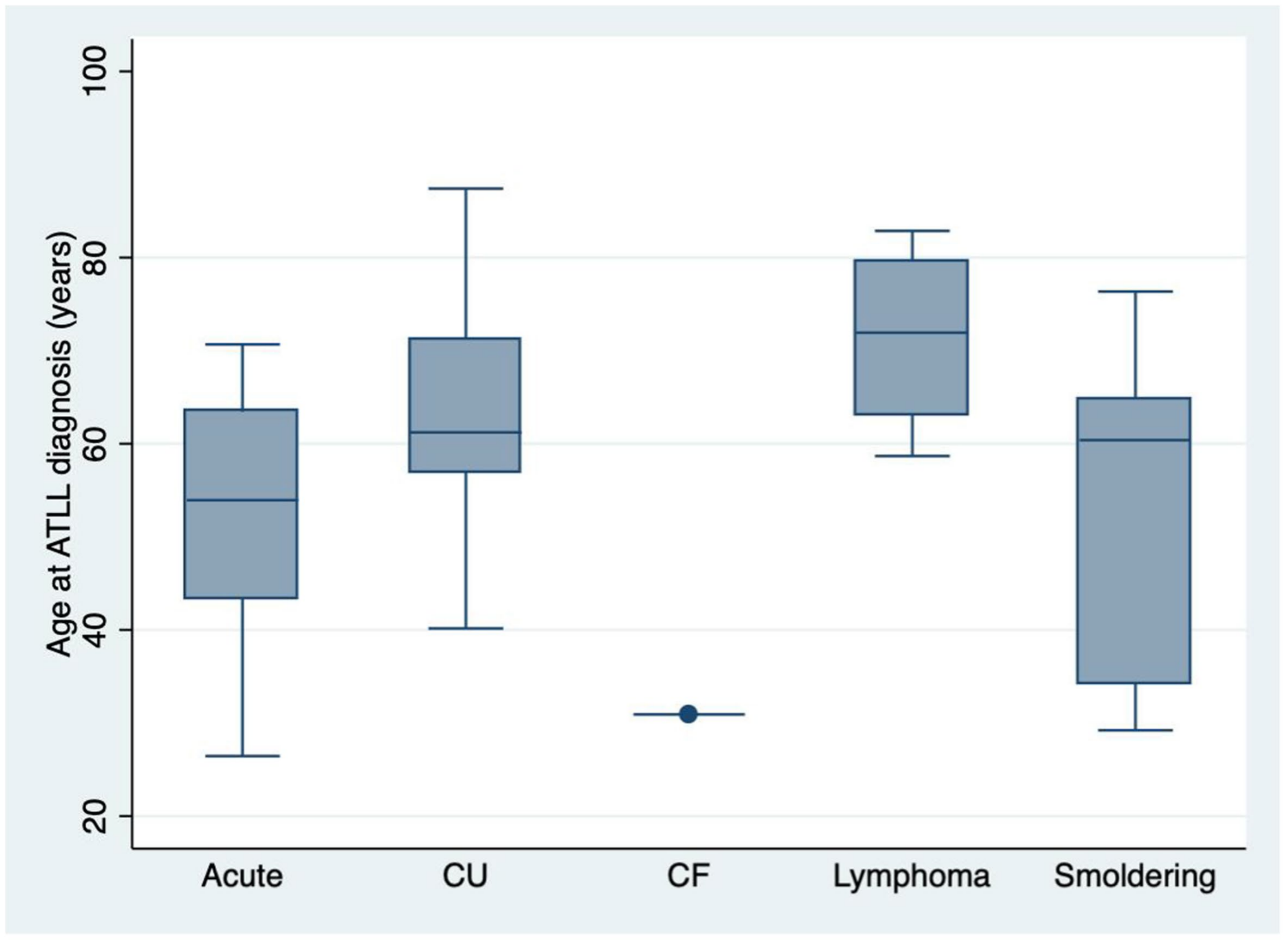
Box plot showing age at diagnosis according to subtypes (*p* = 0.0456). CU: Chronic unfavorable; CF: Chronic favorable. Number of cases = 44.

There was a female predominance, with 24 women and 20 men, and a male:female ratio (M:F) of 0.83, with no statistically significant difference between ATLL subtypes (*p* = 0.406). Age ranged from 26.4 to 87.4 years, with a median age at diagnosis of 59.4 years. Among the ATLL subtypes, lymphoma had older patients at diagnosis (median age of 71.9 years, *p* = 0.0456) (Graph 1).

Regarding ethnicity, 26 patients were white (59%), followed by eight black (18%), seven brown patients (15.9%), and three yellow (6.8%).

Concerning birth location, 17 patients (38.6%) were from the state of São Paulo, two patients (4.5%) from Minas Gerais, and one patient (2.3%) from Rio de Janeiro, totaling 20 patients (45.5%) from the Southeast region. Nine patients (20.5%) were from Bahia, six (13.6%) from Pernambuco, two (4.5%) from Sergipe, one (2.3%) from Alagoas, one (2.3%) from Ceará, one (2.3%) from Piauí and one (2.3%) from Maranhão, totaling 21 patients (47.7%) from the Northeast region. Finally, two patients (4.5%) were from Mato Grosso do Sul and one patient (2.3%) was foreign (Bolivia).

### 3.3 Clinical data

Seven patients (15.9%) had other comorbidities associated with HTLV-1 (before the diagnosis of ATLL): three with infective dermatitis (6.8%) and four with HAM/TSP (9.1%). Among other comorbidities, one patient also had HIV, and another one had hepatitis C infection. Two patients had other cancers: one with urothelial carcinoma of the bladder and the other with colorectal adenocarcinoma, both diagnosed before ATLL.

**Figure 1:**
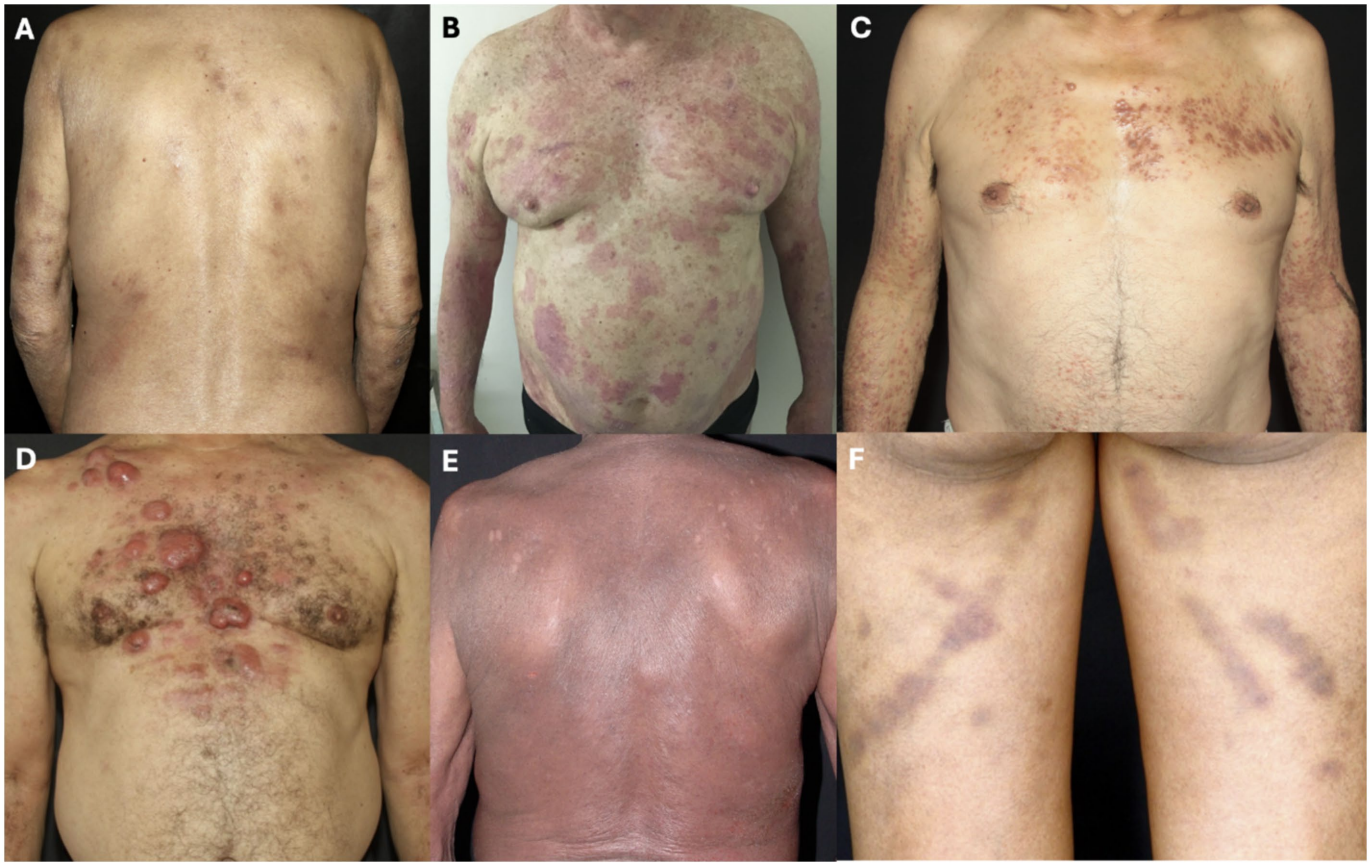
Skin lesions. **(A)** Patches; **(B)** plaques: **(C)** papules; **(D)** nodules and tumors; **(E)** erythroderma; and **(F)** purpuric lesions.

The presence of plaques occurred in 18 patients (40.9%), followed by nodule-tumoral lesions in 15 patients (34.1%), papules in 11 patients (25.0%), erythroderma in 10 (22.7%) and patches in seven patients (15.9%). Other less frequent specific lesions were diffuse ichthyosiform pattern in two patients (4.5%) and purpuric lesions in one patient (2.3%). Other skin conditions observed included palmoplantar hyperkeratosis and subcutaneous panniculitic nodules in two patients each (4.5%), cutis laxa and heliotrope-like periocular lesions in one patient each (2.3%). Figure 1 shows different types of skin lesions observed in the patients of this cohort. More than one type of skin lesion was seen in some patients.

Smoldering ATLL tended to present patches more frequently (40.0%, *p* = 0.075), but there was no statistically significant difference between the ATLL subtype and the other types of skin lesions (Table 2).

Lymph node enlargement was observed in 28 patients (63.6%) clinically and/or through CT or PET tests (Table 2), however, with no statistically significant difference between ATLL subtypes (*p* = 0.298).

### 3.4 Complementary tests

#### 3.4.1 Laboratory tests

Leukocytosis was observed in 23 patients (52.3%), 20 (45.5%) had lymphocytosis >4,000/mL, 34 patients (77.3%) had elevated LDH, six patients (13.6%) had elevated serum calcium levels, five patients (12.2%) had reduced albumin and three patients (6.8%) had increased urea. Beta-2 microglobulin was increased in all 24 patients tested, and results ranged from 1.9 to 10.6 mg/L, with a median of 2.81 mg/L. The presence of flower cells in peripheral blood was observed in six patients (13.6%), five with acute ATLL (35.7% of acute form), and one with lymphoma ATLL (25.0% of lymphoma type) (Table 2, *p* = 0.035).

**GRAPH 2:**
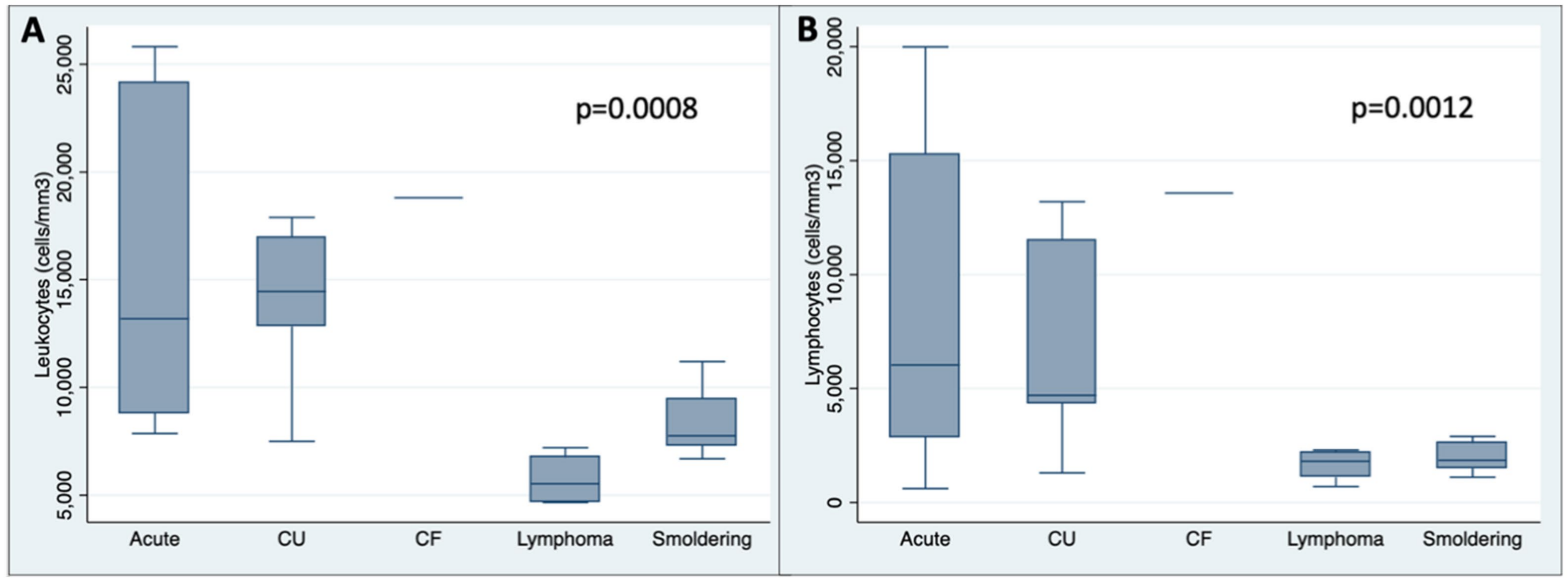
Box plot showing number of leukocytes **(A)** and lymphocytes (cells/mm3) **(B)** among subtypes. CU: Chronic unfavorable; CF: Chronic favorable.

Acute and chronic unfavorable ATLL had leukocytosis and lymphocytosis more frequently, and the total leukocytes and lymphocytes number in those subtypes was higher than the others (Tables 2, 3; Graph 2).

**Table 3:** Laboratory results according to ATLL subtype.

	Acute (IQR)	CU* (IQR)	CF** (IQR^+^)	Lymphoma (IQR)	Smoldering (IQR)	Total (IQR)	*P*-value
Leukocytes (cells/mm^3^)	13,185 (8,800 – 24,140)	14,450 (12,830 – 16,980)	18,810	5,530 (4,710 – 6,750)	7,755 (7,310 – 9,480)	11.145 (7.755–15.980)	0.0008
Lymphocytes (cells/mm^3^)	6,030 (2,880 – 15,280)	4,700 (4,360 – 11,500)	13,580	1,810 (1,110 – 2,200)	1,850 (1,500 – 2,600)	3.055 (1.950–8.385)	0.0012
Urea (mg/dL)	30.5 (26.0–36.0)	34.0 (29.0–41.0)	28.0	39.5 (29.0–48.5)	28.5 (27.0–33.0)	31.0 (27.0–36.5)	0.4488
Albumin (mg/dL)	4.2 (3.9–4.6)	4.1 (3.9–4.3)	4.3	4.1 (3.5–4.2)	4.1 (3.9–4.2)	4.1 (3.9–4.3)	0.6375
Beta-2 microglobulin (mg/dL)	2.8 (2.3–3.0)	2.8 (2.7–3.4)	2.1	7.6	4.6 (1.9–8.9)	2.8 (2.2–3.8)	0.5218
CD4/CD8	10.00 (4.7–14.8)	13.4 (8.3–15.0)	47.0	2.2 (1.4–11.4)	2.2 (1.4–2.8)	8.55 (2.20–14.2)	0.0091
CD4/CD8 ≥ 10 (%)	3/5 (60.0)	8/13 (61.5)	1/1 (100)	1/3 (33.3)	0/8 (0.0)	13/30 (43.3)	0.013
CD4 + CD7-(%)	0.0 (0.0–19.0)	14.5 (0.0–40.0)	92.0	15.0 (7.0–28.5)	15.0 (0.0–20.0)	14.0 (0.0–29.2)	0.4291
CD4 + CD7-≥40%	2/10 (20.0)	4/14 (28.6)	1/1 (100)	1/4 (25.0)	0/7 (0.0)	8/36 (22.2)	0.251
CD4 + CD26-(%)	0.0 (0.0–43.6)	79.5 (0.0–90.3)	92.0	6.0 (0.0–27.0)	30.0 (27.0–30.7)	18.4 (0.0–79.54)	0.3682
CD4+ CD26-≥30%	2/8 (25.0)	5/9 (55.6)	1/1 (100)	0/3 (0.0)	3/5 (60.0)	11/29 (42.3)	0.272

*CU: Chronic unfavorable; **CF: Chronic favorable; ^+^IQR: Interquartile range.

**GRAPH 3:**
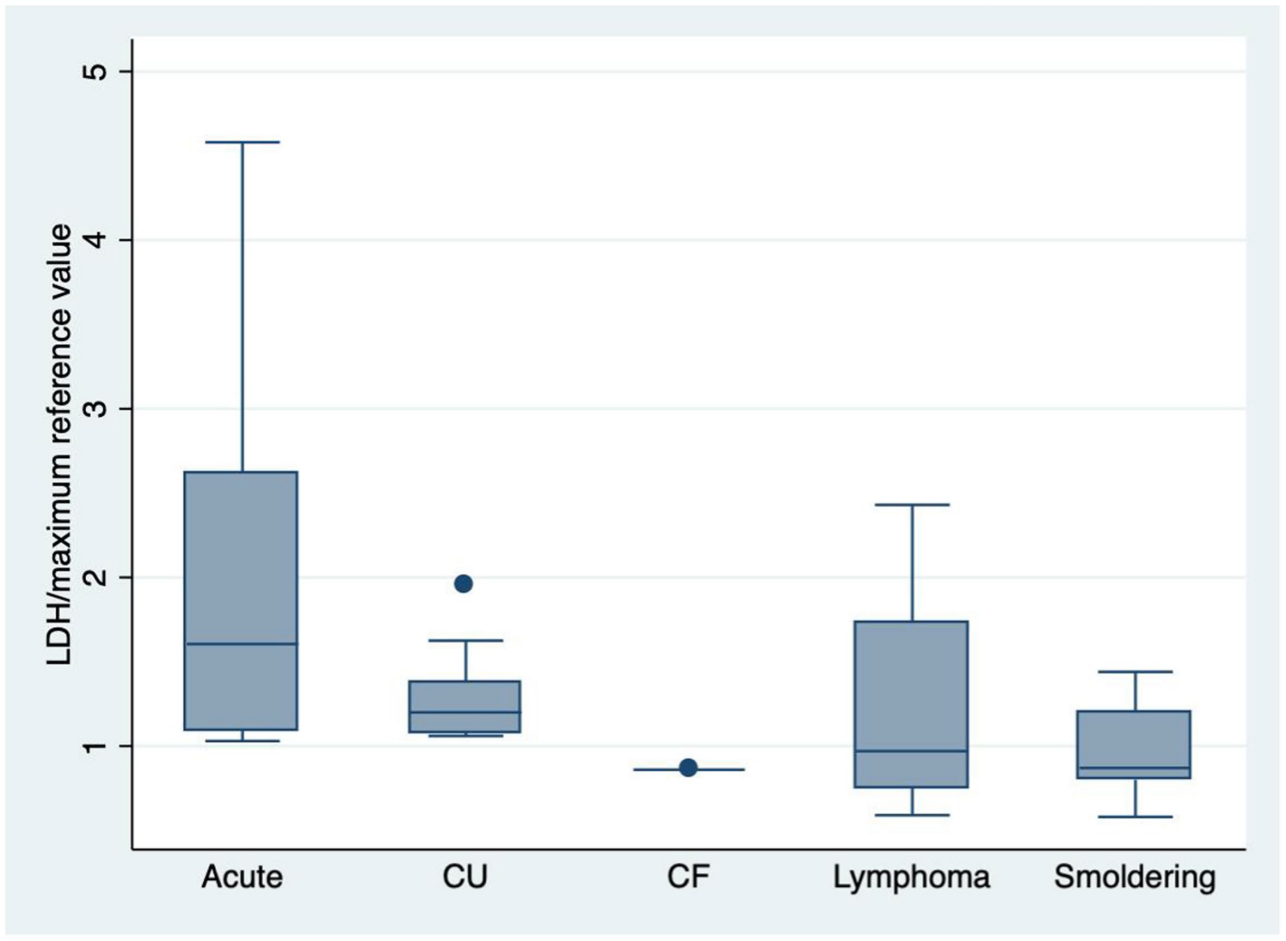
Box plot showing LDH/maximum reference value among subtypes (*p* = 0.0065). CU: Chronic unfavorable; CF: Chronic favorable.

The LDH levels were more frequently increased in the acute and chronic unfavorable subtypes, with statistical significance (*p* < 0.001) (Table 2). The LDH and its maximum reference value ratio were higher in the acute and chronic unfavorable forms (*p* = 0.0065) (Graph 3).

The acute subtype presented hypercalcemia more frequently (46.1%, *p* = 0.007) (Table 2). There was no statistically significant difference between the levels of urea, albumin, or beta-2 microglobulin and subtypes (Table 3). The presence of flower cells was observed in six patients (13.6%), five of 14 patients (35.7%) with acute, and one of four (25.0%) with lymphoma ATLL (Table 2).

#### 3.4.2 Immunophenotyping of lymphocytes by flow cytometry in peripheral blood

Only three patients did not undergo immunophenotyping of lymphocytes by flow cytometry in peripheral blood. All patients presented a CD3 and CD4 positive pattern, CD8 negative or weakly positive. The median CD4/CD8 ratio was 8.5, with higher levels observed in the chronic unfavorable (median of 13.4) and acute (median of 10.0) ATLL (Table 3, *p* = 0.0091). The CD4/CD8 ratio was ≥10 more frequently in the acute (60.0%) and chronic unfavorable (61.5%) subtypes (Table 3, *p* = 0.013).

There was a loss of CD7 in more than 40% of CD4-positive T cells in eight patients (22.2%), with a median loss of 14%. The loss of CD26 in more than 30% of CD4-positive T cells occurred in 11 patients (42.3%), with a median of 18.4% (Table 3). CD25 was assessed in 38 patients, and it was detected in 35 (92.1%) patients, with median expression of 45%, with no statistically significant difference between the groups (*p* = 0.8025).

#### 3.4.3 Clonality of malignant cells

The clonality test for malignant cells in the blood was performed on 30 patients. In 23 (76.7%), a monoclonal population of T-cells was detected. The acute (100%) and the chronic unfavorable (91.7%) subtypes had a monoclonal population on the blood detected more frequently (*p* < 0.001). The clonality testing on the skin was performed on 25 patients, of which 19 (76%) presented a monoclonal population of T-cells. The detection of a monoclonal population on the skin was more frequent in the acute (100%) and lymphoma (100%) forms (*p* = 0.045) (Table 2). In cases with negative or inconclusive T-cell clonality on the skin, the diagnosis of ATLL was confirmed by histopathology and immunohistochemical findings.

#### 3.4.4 Histopathology and immunohistochemical examination

Of the total of 44 patients, three did not present the descriptive histopathology report, only the conclusion of neoplastic cutaneous infiltrate by lymphoma. Of the 41 patients with a descriptive report, 40 (97.6%) presented atypical lymphocytic infiltrate, of which 30 (75%) had epidermal involvement, 33 (82.5%) had dermal involvement, four (10%) had subcutaneous involvement (Table 4). Epidermal involvement was more frequently observed in the smoldering (100%) ATLL (*p* = 0.006). Other histology patterns observed were lichenoid infiltrate in ten (25.0%) patients, folliculotropism in two (5%), androcentrism in two (5%), and perineural involvement in one (2.5%). The presence of Pautrier microabscesses was observed in three patients (7.3%) (*p* = 0.396).

**Table 4:** Histology data according to ATLL subtype.

	Acute	CU*	CF**	Lymphoma	Smoldering	Total	*P*-value
Epidermotropism of lymphocytes (%)	5/11 (45.4)	10/15 (66.7)	1/1 (100)	1/3 (33.3)	8/10 (80.0)	25/40 (62.5)	0.341
Pautrier microabscesses (%)	1/12 (8.3)	1/15 (6.7)	0/1 (0.0)	1/3 (33.3)	0/10 (0.0)	3/41 (7.3)	0.396
Atypical lymphocytes (%)	12/12 (100)	14/15 (93.3)	1/1 (100)	3/3 (100)	10/10 (100)	40/41 (97.6)	0.777
Epidermal involvement (%)	5/11 (45.4)	13/15 (86.7)	1/1 (100)	1/3 (33.3)	10/10 (100)	30/40 (75.0)	0.006
Dermal involvement (%)	10/11 (90.9)	11/15 (73.3)	0/1 (0.0)	3/3 (100)	9/10 (90.0)	33/40 (82.5)	0.242
Subcutaneous involvement (%)	3/11 (27.3)	0/15 (0.0)	0/1 (0.0)	0/3 (0.0)	1/10 (10.0)	4/40 (10.0)	0.233
Lichenoid pattern (%)	2/11 (18.2)	4/15 (26.7)	0/1 (0.0)	1/3 (33.3)	3/10 (30.0)	10/40 (25.0)	0.964
Folliculotropism (%)	0/11 (0.0)	0/15 (0.0)	0/1 (0.0)	0/3 (0.0)	2/10 (20.0)	2/40 (5.0)	0.250
Angiocentrism (%)	0/11 (0.0)	1/15 (6.7)	0/1 (0.0)	0/3 (0.0)	1/10 (10.0)	2/40 (5.0)	0.788
Perineural involvement (%)	0/11 (0.0)	0/15 (0.0)	0/1 (0.0)	0/3 (0.0)	1/10 (130.0)	1/40 (2.5)	0.350
Histologic subtype							
ALCL	1/10 (10.0)	1/13 (7.7)	0/1 (0.0)	1/2 (50.0)	0/7 (0.0)	3/33 (9.1)	0.204
MF	3/10 (30.0)	8/13 (61.5)	1/1 (100)	1/2 (50.0)	6/7 (85.7)	19/33 (57.6)	
PTCL-U	6/10 (60.0)	4/13 (30.8)	0/1 (0.0)	0/2 (0.0)	1/7 (14.3)	11/33 (33.3)	
Loss of CD7	9/9 (100)	11/13 (84.6)	1/1 (100)	2/2 (100)	2/5 (40.0)	25/30 (83.3)	0.082
Lymph node involvement	3/3 (100)	0/2 (0.0)	ND^+^	4/4 (100)	0/1 (0.0)	7/10 (70.0)	0.019

*CU: Chronic unfavorable; **CF: Chronic favorable; ^+^ND: not done.

The IHC analysis showed positivity for CD3 in all 37 (100%) patients tested and for CD4 in 34 patients (97.1%), with one patient with inconclusive CD4 expression. Loss of CD7 was observed in 25 patients (83.3%), with one of these patients presenting total loss. Regarding CD30, it was positive in eight patients (27.6%), another eight (27.6%) had positivity in rare cells, and 13 (44.8%) presented negative results.

The histology and IHC patterns of the samples were divided into MF, ALCL, or PTCL-U. Eleven patients (25%) had insufficient data for classification. Among those who were classified, the MF pattern was observed in 19 patients (57.6%), the ALCL pattern in three patients (9.1%), and PTCL-U in 11 (33.3%) patients (Table 4, *p* = 0.204).

Of the 28 patients with lymph node enlargement detected clinically and/or through CT or PET imaging, ten patients underwent lymph node biopsy, of which three (30%) presented reactive infiltrate and the other seven (70%) presented neoplastic infiltrate. Lymph node involvement was more frequent in the lymphoma (100%) and acute (100%) ATLL (*p* = 0.019).

### 3.5 Treatment

Skin-directed therapies (topical steroids and phototherapy) were performed on 15 patients (36.6%). Patients with acute ATLL were less frequently treated with phototherapy (*p* = 0.051). Zidovudine was used by 21 patients (51.2%), and it was less frequently used by smoldering ATLL patients (*p* = 0.024). IFN was used by 22 patients (53.7%), biological response modifiers (prednisone, acitretin, or mogamulizumab) were used by seven patients (17.1%), and chemotherapy (CT) was performed in 22 patients (53.7%). Radiotherapy (RT) was performed in four patients (9.8%) and one patient (2.4%) underwent allogeneic BMT.

### 3.6 Follow-up

**GRAPH 4:**
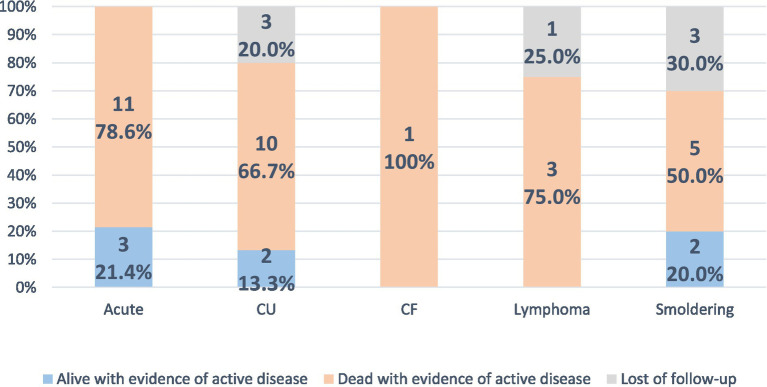
Follow-up data according to ATLL subtype. None of the patients were alive or dead in complete remission at the last evaluation. CU: Chronic unfavorable; CF: Chronic favorable.

During follow-up, most patients died with evidence of active disease at the time of the last evaluation, totaling 30 patients (68.2%). Seven patients were alive with evidence of active disease (15.9%). There was a loss of follow-up of seven patients (15.9%), and none of the patients evaluated were in complete remission at the time of the last evaluation (Graph 4) (*p* = 0.508) (see Table 5).

**Table 5:** Zidovudine and chemotherapy treatments according to ATLL subtype.

Treatment	Acute	CU*	CF**	Lymphoma	Smoldering	Total	*P*-value
Topical steroids (%)	2/13 (15.4)	7/14 (50.0)	0/1 (0.0)	¼ (25.0)	5/9 (55.6)	15/41 (36.6)	0.189
Phototherapy (%)	1/13 (7.7)	7/14 (50.0)	1/1 (100)	2/4 (50.0)	4/9 (44.4)	15/41 (36.6)	0.051
Zidovudine (%)	6/13 (46.1)	10/14 (71.4)	1/1 (100)	3/4 (75.0)	1/9 (11.1)	21/41 (51.2)	0.024
Interferon (%)	7/13 (53.8)	9/14 (64.3)	1/1 (100)	3/4 (75.0)	2/9 (22.2)	22/41 (53.7)	0.226
Biologic response modifiers	8/13 (61.5)	10/14 (71.4)	1/1 (100)	3/4 (75.0)	4/9 (44.4)	26/41 (63.4)	0.718
Chemotherapy (%)	8/13 (61.5)	6/14 (42.9)	1/1 (100)	3/4 (75.0)	4/9 (44.4)	22/41 (53.7)	0.682

*CU: Chronic unfavorable; **CF: Chronic favorable.

**Figure 2:**
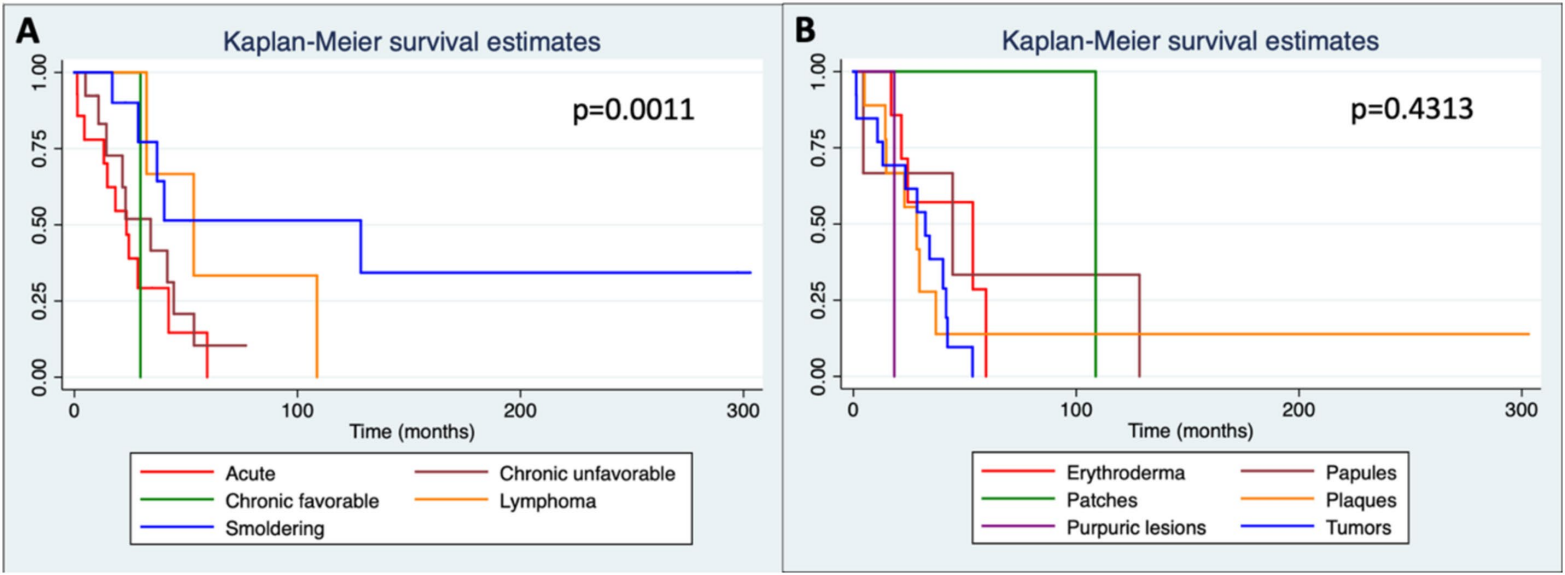
Kaplan–Meier curves showing overall survival in months according to each subtype **(A)** and to skin lesions **(B)**. Patches (*n* = 7); plaques (*n* = 18); Papules (*n* = 11); nodule/tumors (*n* = 15); erythroderma (*n* = 10); purpuric lesions (*n* = 1).

The median overall survival was 32.3 months. It was 23.3 months in the acute form, 34.1 months in the chronic unfavorable, 53.5 months in the lymphoma, 128.4 in the smoldering, and the patient with chronic favorable died after 29.6 months of the diagnosis. The acute form presented lower, and the smoldering form had higher median overall survival (*p* = 0.0011) (Figure 2; Table 2).

Survival according to the types of skin lesions was higher among patients with patches (median survival of 108.7 months) compared to those with erythroderma (53.6 months), papules (44.5 months), nodule-tumoral lesions (32.3 months), and plaques (28.4 months). However, there was no statistically significant difference between the types of lesions and survival (*p* = 0.4313) (Figure 2).

### 3.7 Univariate and multivariate analysis

The results of the univariate and multivariate analyses are described in Tables 6–9.

**Table 6:** Cox proportional hazards analysis of clinical factors (univariate analysis, controlled for sex and age).

Univariate analysis				
		Hazard Ratio	95% confidence interval	*p*-value
Age		1	0.98–1.03	
Gender	Female	1		
	Male	1.33	0.63–2.85	0.449
Subtype	Acute	1		
	Chronic unfavorable	0.66	0.27–1.60	0.356
	Chronic favorable	0.93	0.12–7.34	0.942
	Lymphoma	0.38	0.10–1.44	0.156
	Smoldering	0.19	0.06–0.65	0.008
Ethnicity	Yellow	1		
	White	1.21	0.28–5.26	0.801
	Black	1.16	0.22–6.02	0.862
	Brown	1.45	0.26–8.04	0.672
HTLV-1-associated comorbidities	No	1		
	Yes	0.58	0.19–1.75	0.338
Patches	No	1		
	Yes	0.41	0.12–1.42	0.16
Plaques	No	1		
	Yes	1.92	0.9–4.1	0.092
Papules	No	1		
	Yes	1.83	0.85–3.96	0.124
Nodules/tumors	No	1		
	Yes	2.11	0.95–4.70	0.067
Erythroderma	No	1		
	Yes	0.71	0.28–1.75	0.451
Purpuric lesions	No	1		
	Yes	3.62	0.46–28.64	0.222

**Table 7:** Cox proportional hazards analysis of laboratory data (univariate analysis, controlled for sex and age).

Univariate analysis				
		Hazard Ratio	95% confidence interval	*P*-value
Leukocytes		1.03	1.01–1.05	0.011
Leukocytosis	No	1		
	Yes	1.76	0.82–3.85	0.142
Lymphocytes		1.04	1.01–1.07	0.011
Lymphocytosis	No	1		
	Yes	1.86	0.87–3.97	0.108
Eosinophils		1.23	0.43–3.50	0.5276
Eosinophilia	No	1		
	Yes	0.89	0.35–2.24	0.798
LDH/reference		1.27	0.81–2.00	0.303
Increased LDH	No	1		
	Yes	1.95	0.78–4.89	0.152
Calcium levels		1.18	0.89–1.57	0.246
Hypercalcemia	No	1		
	Yes	1.15	0.39–3.35	0.799
Urea		1.04	1.01–1.07	0.008
Increased urea	No	1		
	Yes	3.14	0.92–10.76	0.068
Albumin		1.11	0.56–2.17	0.768
Hypoalbuminemia	No	1		
	Yes	0.95	0.22–4.13	0.948
HIV	No	1		
	Yes	0.98	0.13–7.40	0.987
CD4/CD8		0.99	0.95–1.04	0.905
CD4/CD8 ≥ 10	No	1		
	Yes	0.96	0.37–2.51	0.932
CD4 + CD7-		1.01	0.99–1.02	0.491
CD4 + CD7- ≥ 40%	No	1		
	Yes	1.14	0.42–3.10	0.792
CD4 + CD26-		1.01	0.99–1.02	0.383
CD4 + CD26- ≥ 30%	No	1		
	Yes	2.25	0.76–6.62	0.141
Skin clonality	Polyclonal	1		
	Monoclonal	3.21	0.70–14.66	0.132
Blood clonality	Polyclonal	1		
	Monoclonal	1.39	0.46–4.18	0.561

**Table 8:** Cox proportional hazards analysis of histology data (univariate analysis, controlled for sex and age).

Univariate analysis				
		Hazard ratio	95% confidence interval	*p*-value
Lymph node biopsy	Reactional	1		
	Involved	1.42	0.27–7.45	0.678
Infiltrate in epidermis	No	1		
	Yes	0.49	0.21–1.13	0.094
Infiltrate in dermis	No	1		
	Yes	0.91	0.27–3.09	0.881
Infiltrate in subcutaneous	No	1		
	Yes	1.07	0.31–3.67	0.916
Lichenoid infiltrate	No	1		
	Yes	0.64	0.26–1.60	0.343
Small lymphocytes	No	1		
	Yes	0.64	0.22–1.90	0.423
Medium lymphocytes	No	1		
	Yes	0.97	0.35–2.67	0.947
Large lymphocytes	No	1		
	Yes	1.39	0.47–4.14	0.555
Epidermotropism of lymphocytes	No	1		
	Yes	0.71	0.32–1.58	0.397
Pautrier’s microabscesses	No	1		
	Yes	0.73	0.17–3.15	0.672
Loss of CD7	No	1		
	Yes	0.81	0.23–2.89	0.75
Histologic type	MF	1		
	ALCL	3.09	0.66–14.58	0.153
	PTCL-U	1.48	0.62–3.55	0.380
Lymph node biopsy	Reactional	1		
	Involved	1.42	0.27–7.45	0.678

**Table 9:** Cox proportional hazards analysis of clinical, histological, laboratory factors and overall survival (multivariate analysis, controlled for sex and age).

Multivariate analysis			
	Hazard ratio	95% confidence interval	*p*-value
Subtype	0.72	0.56–0.92	0.009
Leukocytes	1	0.99–1.01	0.994
Lymphocytes	1.01	0.99–1.01	0.928
Urea	1.04	1.00–1.08	0.019

In the univariate analysis, factors associated with poorer prognosis included acute form, leukocytes, lymphocytes, and urea levels. After multivariate analysis (Table 9), only the Shimoyama classification and urea levels were associated with higher risk of mortality.

## 4 Discussion

This study presented a significant number of patients with ATLL and specific cutaneous manifestations. Another study conducted in Bahia, Brazil, and published in 2007 analyzed 70 patients with ATLL, of which 47 had cutaneous involvement (42). In the world, only one study conducted in Japan evaluated a larger sample of 119 patients with ATLL and cutaneous lesions (30); another study conducted in France also evaluated patients with ATLL and specific cutaneous manifestations but with a smaller sample (37 patients) (43).

It is known that ATLL is observed mainly in adults, with an average age at diagnosis that may vary according to the country, but which is around 60 years of age (20, 30, 42, 44–47). In Brazil, studies demonstrate an earlier average age at diagnosis of around 50 years (19, 42), but our study presented a median compatible with other countries (60 years).

Regarding gender, there was a predominance of females in our sample (M:F ratio of 0.83), while in most studies there is no gender predominance or a slight male predominance (ratio of 1.1:1) (30, 45, 48).

We observed a predominance of patients from the Northeast region of Brazil (47.7%), especially from Bahia, even though they are patients treated at a hospital located in the city of São Paulo. This data reflects the high prevalence of HTLV-1 infection in Bahia, considered an endemic region of the virus due to the African ancestry of its population. In addition, this data also reflects the need for knowledge of HTLV-1 and diseases related to it even in non-endemic areas, due to migratory phenomena (1, 13–15).

The ethnicity of patients with ATLL reflects the regions of the world in which the HTLV-1 virus is most present, with a predominance of black patients of African ancestry and Asian patients of Japanese origin. In our study, however, there was a higher prevalence of white patients (59%). We believe that this data, which conflicts with the literature, is caused by the great miscegenation in the Brazilian population, which makes ethnicity difficult to classify. Furthermore, in our study, ethnicity was self-declared in the medical records, which also represents another confounding factor in the characterization of individuals (3, 19, 42, 45, 48).

Concerning classification, the chronic subtype was the most frequent (34.1%), followed by acute (31.8%) and smoldering (22.7%), which differs from the literature that indicates the acute, lymphoma, or smoldering forms as the most common (20, 30, 42, 43). This difference can be justified by the fact that the chronic form more frequently presents specific skin lesions, which are the object of our study (29, 42, 43). In addition, patients with the acute and lymphoma type forms tend to have a worse prognosis with rapid clinical deterioration and are most often followed up by hematologists.

The specific skin lesions were variable and consistent with what is described in the literature. However, in most studies, the most frequent lesions were nodule-tumoral, while in our study the most frequent were plaques in 40.9% of cases (Table 10) (29, 30, 42, 43, 49, 50). Since in the present study, the chronic form was the most common, it can be inferred that this was the reason why nodule-tumoral lesions (more frequently observed in the more aggressive forms) were not the most frequent (30). It is also important to note that we only had one case of purpuric type, which is compatible with the literature since it is the rarest specific cutaneous manifestation of ATLL (29, 30, 42, 43, 49, 50).

**Table 10:** Distribution of specific skin lesions compared with other studies.

	Patches	Plaques	Papules	Nodules tumors	Erythroderma	Purpuric	Ichthyosis like	Total
Our study, Southeast, Brazil	7 (15.9%)	18 (40.9%)	11 (25.0%)	15 (34.1%)	10 (22.7%)	1 (2.3%)	2 (4.5%)	64
Bittencourt et al. (42) Brazil	8 (6.7%)	32 (26.9%)	23 (19.3%)	46 (38.7%)	5 (4.2%)	5 (4.2%)	0	119
Sawada et al. (30) Japan	2 (11.8%)	4 (23.5%)	4 (23.5%)	6 (35.3%)	1 (5.9%)	0	0	17
Higuchi et al. (49) Japan	4 (14.8%)	7 (25.9%)	6 (22.2%)	8 (29.6%)	0	2 (7.4%)	0	27
Hurabielle et al. (43) France	2 (2.7%)	19 (26%)	18 (24.7%)	14 (19.2%)	20 (27.4%)	0	0	73
Marchetti et al. (29) USA	5 (17.2%)	8 (27.6%)	5 (17.2%)	9 (31%)	2 (6.9%)	0	0	29
Pezeshkpoor et al. (50) Iran	0	2 (8.7%)	16 (69.9%)	2 (8.7%)	1 (4.3%)	0	2 (8.7%)	23
Total	28 (7.9%)	90 (25.6%)	83 (23.6%)	100 (28.4%)	39 (11.1%)	8 (2.3%)	4 (1.1%)	352

*Other lesions in our study: six patients (palmoplantar hyperkeratosis and panniculitic subcutaneous nodules in two patients each, cutis laxa, and heliotrope-like periocular lesions in one patient each).

Regarding laboratory data, attention was drawn to the altered Beta-2 microglobulin dosage in all patients in whom this test was performed, with an increased result that ranged from 1.9 to 10.6 mg/L (reference value: 1–1.7 mg/L) and a median of 2.81 mg/L. However, Beta-2 microglobulin levels were not associated with a higher risk of death, in contrast to other types of cutaneous lymphomas such as MF and SS, where its increase indicates a worse prognosis (51).

In the acute form, the number of leukocytes and lymphocytes was higher than in the other subtypes (*p* = 0.0008 and *p* = 0.0012 respectively), which demonstrates and corroborates the leukemization seen in the acute form (20).

Increased LDH levels and a higher ratio of LDH and its maximum normal value were more frequently observed in the acute and chronic unfavorable subtypes. This finding is consistent with what is seen in the literature since increased LDH represents a worse prognosis and is one of the criteria for distinguishing between chronic favorable and unfavorable forms (20, 28).

Regarding immunophenotyping of peripheral blood by flow cytometry, all patients who underwent this test presented a CD3 and CD4 positive pattern, results compatible with a CD4 positive T-cell lymphoma (44). The CD25 was evaluated in 38 patients in peripheral blood with positivity in 92.1% of them. Although the positivity of this marker in cases of ATLL is high, it is not a specific test (20, 31). The CD4/CD8 ratio and evaluation of the percentage of CD4 + CD7-and CD4 + CD26-cells are used as diagnostic criteria for Sézary syndrome (52). In our study, we observed CD4/CD8 ≥ 10 more frequently in the acute and chronic unfavorable forms (*p* = 0.013), reflecting the degree of peripheral blood involvement in these subtypes. However, there is no data in the literature on the use of these criteria in ATLL. We believe that, since the neoplastic cell of ATLL may present loss of CD7 and CD26 markers and an increase in the CD4/CD8 ratio, studies with a larger number of patients and evaluation of flow cytometry could define more objective diagnostic criteria and even evaluate a possible correlation of these markers with the prognosis.

The histology and IHC pattern of the samples was compatible with MF in 19 patients (57.6%), with ALCL in 3 patients (9.1%), and with PTCL-U in 11 (33.3%) patients. The study that proposed the division according to these patterns found a different result, with 67% PTCL-U pattern, 22.8% MF pattern, 5.7% ALCL pattern, and 4.3% with leukemic pattern (cases of the acute form with hematological diagnosis). We believe this divergence occurred due to the classification of patients in this other study, in which there was a higher frequency of acute (38.6% compared to 31.8% in our study) and lymphoma (38.6% compared to 9.1% in our study) subtypes (42).

The treatments administered to the patients in our study were consistent with the protocols recommended in Brazil. Skin-directed therapies (topical corticosteroids and phototherapy), AZT/IFN, biological response modifiers (prednisone, acitretin, and mogamulizumab), CT, RT, and allogeneic BMT were administered. Mogamulizumab is not available in Brazil, but it was used by one patient during a research protocol, without a satisfactory response. In the comparison between the subtypes, the acute form tended to use less frequent phototherapy (*p* = 0.051), which is justifiable since it is a more aggressive subtype, with significant hematologic involvement, requiring systemic therapies. On the other hand, zidovudine was less frequently used in smoldering ATLL, because it is a more indolent disease, usually treated with “watch and wait” or skin-directed therapies. No statistically significant difference was observed correlating the other treatments and subtypes (33, 35, 37).

The overall mortality found in the study was 68.2%, which reflects the high aggressiveness of ATLL. Furthermore, as expected, no patient evolved with complete remission of the disease during the follow-up; the only patient who underwent allogeneic BMT presented relapse after 2 years and died due to disease progression (33).

Among the subtypes, the median overall survival was lower in the acute form (23.3 months), which is also consistent with data in the literature, since this is the most aggressive form of the disease (20, 22, 26). The number of patients with the favorable chronic form was small (one patient), which makes the comparison with the chronic unfavorable form difficult.

The presence of skin lesions in patients with ATLL was associated with a worse prognosis compared with patients without skin lesions (30, 31). A study published in 2011 observed, after multivariate analysis, that patients with erythroderma and nodule-tumoral lesions had a worse prognosis and configured an independent prognostic factor for ATLL (30). However, we did not observe an association between the type of skin lesion and prognosis.

After uni and multivariate analysis, only acute Shimoyama classification and higher urea levels were associated with poorer prognoses.

Limitations of this study include its retrospective nature. Evaluation of skin lesion response is difficult because many cases did not have photos during the evolution or at the last evaluation, only at diagnosis. Also, some clinical and laboratory data were missing. The number of patients is small to reach statistical significance in multiple analyses, however, considering the rarity of this disorder, it is a significant number and may add to the literature since we reported the experience of a large tertiary center in Brazil.

## 5 Conclusion

We described demographic, clinical, histopathology, laboratory, and follow-up data of a large cohort of ATLL patients with specific skin lesions in Brazil. The Northeast of the country is endemic for HTLV-1 infection, and most patients evaluated in this study were born in this region. The clinical, histopathology, and laboratory differences between this study and the literature may be due to the different frequencies of ATLL subtypes. Since we evaluated only ATLL patients with specific skin lesions at a Dermatology service, some patients with more aggressive types of ATLL (acute and lymphoma type) may have been followed only in the Hematology department. Thus, prospective multicentric studies are necessary to identify factors associated with prognosis and to better classify the patients according to Shimoyama criteria.

## Data Availability

The original contributions presented in the study are included in the article/supplementary material, further inquiries can be directed to the corresponding author.
